# Effect of Isoproterenol on LDL Susceptibility to Oxidation and Serum Total Antioxidant Capacity in Cyclosporine-Treated Rats

**Published:** 2010-08-01

**Authors:** H. Foroughimoghaddam, A. Ghorbanihaghjo, N. Rashtchizadeh, H. Argani

**Affiliations:** 1 *I.A.U. Tabriz Branch, Young Researchers Club, Tabriz, Iran, *; 2 *Drug Applied Research Center, Tabriz University of Medical Sciences, Tabriz 51664, Iran, *; 3 *Biotechnology Research Center, Tabriz University of Medical Sciences, Tabriz 51664, Iran, *; 4 *Shahid Beheshti University of Medical Sciences, Tehran, Iran*

**Keywords:** Isoproterenol, Cyclosporine, LDL Susceptibility to Oxidation, Total Antioxidant, Rat

## Abstract

Background: Cyclosporine therapy is associated with a variety of adverse effects. Recent studies have suggested increased oxidative stress as a cause of these side effects.

Objective: Since, melatonin is one of the most powerful known antioxidants, and considering that isoproterenol is one of the drugs stimulating endogenous melatonin production, we tried to determine the effect of isoproterenol on LDL susceptibility to oxidation and serum total antioxidant capacity in cyclosporine-treated rats.

Methods: 32 male Wistar rats were divided into four groups: group A were controls that received placebo; group B received intraperitoneal isoproterenol (20 mg/kg/d) alone; group C received intravenous cyclosporine (15 mg/kg/d) alone; and group D received both drugs simultaneously at the same doses and durations—cyclosporine one week after administration of isoproterenol. Blood samples were drawn four times from rats in each group: before injections, during the treatment, end of the treatment, and one week after the last injections.

Results: There was a significant (p<0.05) increase in LDL susceptibility to oxidation, and a decrease in serum total antioxidant capacity (p<0.05) in group C rats. But, there were no significant changes in group B and D rats in terms of LDL susceptibility to oxidation and total antioxidant capacity.

Conclusion: Isoproterenol may be capable of delaying adverse effects of cyclosporine by preventing the increase in LDL susceptibility to oxidation, and decrease in serum total antioxidant capacity.

## INTRODUCTION

Over the past several years, cyclosporine (CsA) has been used to prevent rejection of transplanted organs. Nevertheless, its adverse effects restrict its clinical application [1-3]. Chronic administration of CsA produces renal injury, which is termed chronic CsA nephropathy, and is thought to be a major side effect and is one of the known nonimmunological factors causing 30%–50% of chronic allograft nephropathy in the total renal transplant population [4]. Since CsA is the prime agent used in immunosuppressive therapy, lack of efficacy for the prevention of long-term allograft failure may be due to the side effects of CsA [5]. Clinical and experimental data strongly suggest that CsA-induced nephrotoxicity results from increased production of free radicals exclusive to the kidney [6,7]. As oxidative stress resulting from CsA administration may be responsible for its adverse effects, antioxidant therapy and usage of protective agents to reduce CsA-induced free oxygen radicals were of important therapeutic purposes for researchers focusing on CsA-treated patients [6,8]. Usage of spironolactone, calcium channel blockers, and antioxidant agents such as ramipril, allopurinol and melatonin have resulted in some desirable outcomes [9-11]. 

Melatonin (mainly secreted by the pineal gland) has been reported to have antioxidant properties in addition to its known hormonal activities. It is an effective scavenger of both the highly toxic hydroxyl and peroxyl radicals. Additionally, melatonin may stimulate antioxidant enzymes such as superoxide dismutase and glutathione peroxidase, and reduce lipid peroxidation [12,13]. Melatonin hormone plays an important role in protecting cells from free radicals damage [14]. Aministration of melatonin can reduce the level of oxidation metabolites of DNA, lipids, proteins and amino acids [15-17]. Exogenous melatonin exerts a protective effect on CsA-induced nephrotoxicity in rat [8]. But, the question is that can we suppress the activity of oxygen free radicals by stimulating endogenous melatonin production? 

There are a few studies around drugs capable of stimulating biosynthesis and secretion of melatonin, and some limited agents like epinephrine and isoproterenol has been discussed [18, 19]. Thus, we tried to investigate effect of isoproterenol on CsA-treated rats in terms of LDL susceptibility to oxidation, and serum total antioxidant capacity. 

LDL oxidation process consists of three phases: 1) lag phase, including reactions which result in production of lipid peroxides and peroxyl radicals; 2) propagation phase, which includes different reactions which result in rapid increase of radicals in the environment, and 3) decomposition phase, which results in decomposition of unsaturated fatty acids and Apo(B)_100_ protein, and oxidative changes of cholesterol [20-25].

## MATERIALS AND METHODS:

According to the Guide for Care and Use of Laboratory Animals (DHEW Publication No. 78-23, NIH revised 1978) and local guidelines for humane use of animals in research, we housed 32 young male Wistar rats weighing 225 to 280 g three per cage, with *ad libitum *access to compact food and water containing all essential ingredients, including vitamins and minerals. Animals were kept under similar laboratory conditions (18 °C to 23 °C room temperature) with alternating 12-hour cycles of light and dark.

After one week of maintenance in a standard environment for conditioning, the animals were randomly allocated into four study arms: group A controls (n=8) received no interventions but daily intraperitoneal, and intravenous (via the tail vein) injections of normal saline as placebo; group B (n=8) rats received for 21 days intraperitoneal injections of isoproterenol (DL-isoproterenol hydrochloride; 20 mg/kg/d); group C (n=8) rats received intravenous CsA (15 mg/kg/d) injections via the tail vein for 14 days without isoproterenol; and group D (n=8) rats received both drugs—CsA (15 mg/kg/d similar to group C) was administered one week after start of premedication with isoproterenol (20 mg/kg/d intraperitoneally) and continued for 14 days.

Blood samples were collected under ether anesthesia via the orbital sinus using a fine-walled pipette in four stages for each group: 1) at the beginning of the experiment just prior to drug administration; 2) during the drug administration protocol (10^th^ day for groups A, B and D, and 7^th^ day for group C); 3) at the end of the treatment; and 4) one week after the treatment. Serum separated by centrifugation for 15 min at 3000 rpm was stored at 80 °C until analysis. 

Serum melatonin concentration was determined by a commercially available enzyme-linked immunoassay (ILB Co), and total antioxidant capacity in serum samples by a spectrophotometric method (Randox kits). LDL susceptibility to oxidation was evaluated by the formation of conjugated dienes induced by copper (Cu^++^). LDL was separated from plasma by ultracentrifugation (Beckman Optima TLX) with a gradient of discontinued density in a vertical column. The isolated LDL was dialyzed overnight at 4 ºC against a 25 mM phosphate buffer, pH 7.2, and 0.1 M NaCl. The LDL (100 mg protein/mL) was then incubated in the presence of 10 µM CuSO_4_ at 37 ºC. LDL oxidation kinetics was continuously monitored by measuring the conjugated diene formation, with the increase in absorbancy at 234 nm. The absorbance was analyzed at 10-min intervals (Cecil 8000 Spectrophotometer). The presentation of results is figured by the lag phase (reflecting the resistance of LDL to oxidation, measured in min) directly related to the amount of antioxidant carried by the LDL. A longer lag phase demonstrates a reduced susceptibility of LDL particles to oxidation, caused by a higher antioxidant concentration, whereas a shorter lag phase means that LDL particles take less time to oxidize, due to the lower presence of antioxidants [26-29]. 

Data are expressed as mean values. The Wilcoxon signed-rank test was used to assess the significance of differences between basal measurements and one week after the treatment. Comparisons between the basal, during the treatment, and end of the treatment measurements were evaluated using the Friedman test. All analytical tests were performed by SPSS. A p value <0.05 was considered statistically significant.

## RESULTS

Comparison of serum melatonin, lag phase, and total antioxidant capacity levels measured in the first stage of sampling in all groups (Table 1) demonstrated that there was no significant difference in serum melatonin level (p>0.7), lag phase (p>0.5), and total antioxidant capacity (p>0.9) in all groups at the beginning of the experiment (Kruskal-Wallis test). Also, there was no significant difference in changes of said values in control group by time (Table 1). 

**Figure 1:**
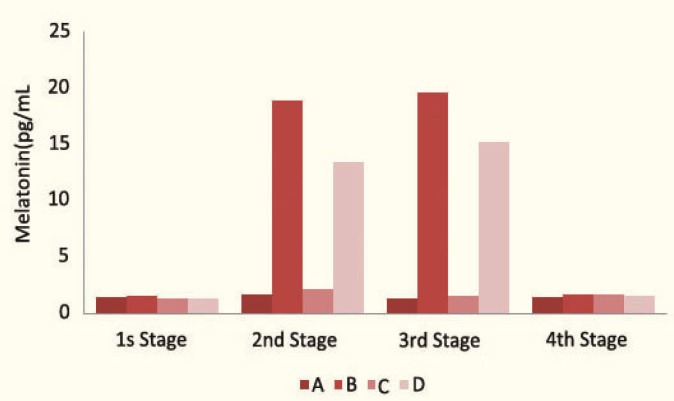
Melatonin concentrations in 4 stages of sampling in the study groups:

Administration of isoproterenol had significantly (p<0.05) increased mean serum melatonin level in group B and D rats (Table 1 and Fig. 1). 

**Table 1:** Mean values of melatonin (pg/mL), lag phase (min), and total antioxidant capacity (mM/L) in the four stages of sampling in the study groups

	**Group A**			**Group B**			**Group C**			**Group D**		
	Melatonin	Lag Phase	Total Antioxidant	Melatonin	Lag Phase	Total Antioxidant	Melatonin	Lag Phase	Total Antioxidant	Melatonin	Lag Phase	Total Antioxidant
**1** ^st^ Stage	1.37	122.0	1.30	1.51	123.9	1.34	1.31	127.3	1.29	1.35	122.9	1.27
**2** ^nd^ Stage	1.63	123.9	1.26	18.91	134.1	1.38	2.15	121.9	1.15	13.51	123.0	1.24
**3** ^rd^ Stage	1.35 ‡P = 0.368	126.0 ‡P = 0.798	1.25 ‡P = 0.908	19.67 ‡P = 0.002٭	119.1 ‡P = 0.044٭	1.52 ‡P = 0.417	1.49 ‡P = 0.197	113.0 ‡P = 0.005٭	0.60 ‡P = 0.005٭	15.19 ‡P = 0.002٭	123.6 ‡P = 0.607	1.20 ‡P = 0.417
**4** ^th^ Stage	1.39 §P = 0.779 †P = 0.944	125.4 §P = 0.944 †P = 0.620	1.27 §P = 1.000 †P = 0.944	1.62 §P = 0.012٭ †P = 0.528	121.4 §P = 0.262 †P = 0.866	1.45 §P = 0.674 †P = 0.528	1.61 §P = 0.726 †P = 0.207	116.6 §P = 0.400 †P = 0.017٭	0.93 §P = 0.068 †P = 0.025٭	1.49 §P = 0.012٭ †P = 0.401	114.6 §P = 0.068 †P = 0.093	1.20 §P = 0.865 †P = 0.674

A: Control group

B: Only isoproterenol treatment

C: Only CsA treatment

D: CsA + isoproterenol treatment

1^st^ Stage: basal measurements

2^nd^ Stage: during the treatment

3^rd^ Stage: end of the treatment

4^th^ Stage: a week after the treatment

‡ Comparison was made between base, the second stage, and the third stage of sampling values in each group by Friedman test.

§ Comparison was made between the third stage and the fourth stage of sampling values in each group by Wilcoxon test.

† Comparison was made between base and the fourth stage of sampling values in each group by Wilcoxon test.

* Statically significant (p<0.05)

**A:** control group

**B:** Only isoproterenol treatment

**C:** Only CsA treatment

**D:** CsA + isoproterenol treatment

**1st Stage:** basal measurements

**2nd Stage:** during the treatment

**3rd Stage:** end of the treatment

**4th Stage:** a week after the treatment

**Figure 2:**
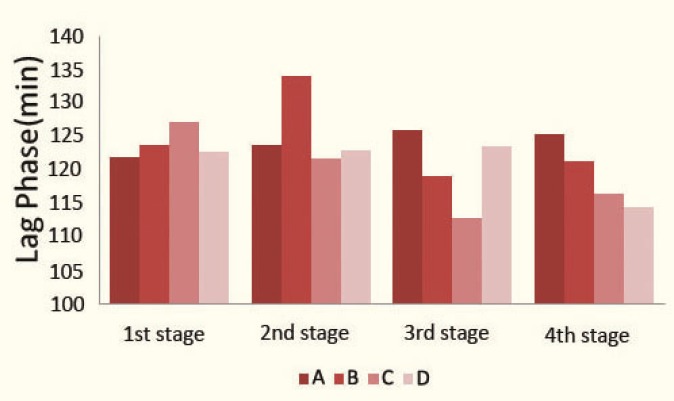
Lag phase in 4 stages of sampling in the study groups:

There was a significant difference (p=0.044) in changes of lag phase in group B rats (Friedman test), but, because these changes were not linear, we decided to perform other analyses in which we compared the measured amounts in the beginning of the experiment (the first stage), during the treatment (the second stage), and end of the treatment (the third stage) with each other using Wilcoxon test; there was no significant difference (p=0.123) in comparison of the measured amounts of the first stage and the second stage, nor in the measured amounts of the first stage and the third stage (p=0.326), but only a significant decrease in comparison of the measured amounts of the second stage and the third stage (p=0.017); that is to say, isoproterenol did not have a noticeable influence on changes of LDL susceptibility to oxidation in group B rats. There was no significant changes in measured lag phase in group D rats (p=0.607), as well. But, CsA administration significantly decreased (p=0.005) the lag phase in group C rats (Table 1 and Fig. 2). 

**A:** control group

**B:** Only isoproterenol treatment

**C:** Only CsA treatment

**D: **CsA + isoproterenol treatment

**1st Stage:** basal measurements

**2nd Stage:** during the treatment

**3rd Stage:** end of the treatment

**4th Stage:** a week after the treatment

**Figure 3:**
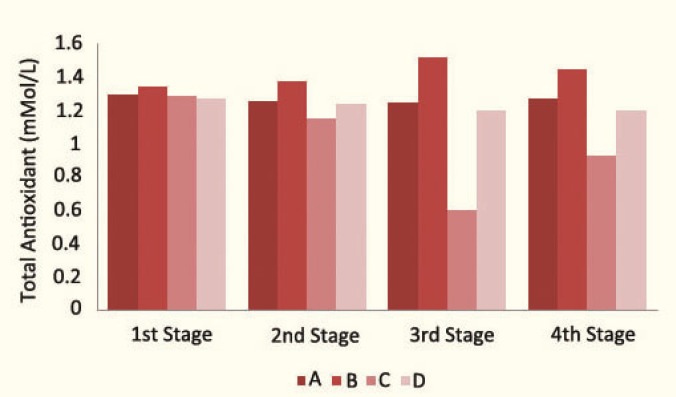
Total antioxidant capacity in 4 stages of sampling in the study groups

There were no significant changes in groups B and D, but a statically significant decrease (p=0.005) in group C rats in terms of total antioxidant capacity (Table 1 and Fig. 3). 

**A:** control group

**B:** Only isoproterenol treatment

**C:** Only CsA treatment

**D:** CsA + isoproterenol treatment

**1st Stage:** basal measurements

**2nd Stage:** during the treatment

**3rd Stage:** end of the treatment

**4th Stage:** a week after the treatment

One week after the drug withdrawal in group C rats, LDL susceptibility to oxidation was still higher (p=0.017), and the total antioxidant capacity was still lower (p=0.025) than their initial levels before administration of CsA. Also, there was a rapid and significant return in measured melatonin concentration, but not a significant change in the lag phase and total antioxidant capacity in groups B and D (Table 1). 

## DISCUSSION

The present investigation revealed that administration of CsA (15 mg/kg/d for 14 days) resulted in a significant increase in LDL susceptibility to oxidation, and decrease in serum total antioxidant capacity. So far, several papers have reported data that shows such adverse effects for CsA as well [30-33]. It is obvious that these adverse effects limit CsA clinical use. Consequently, there is much interest in developing new methods to do away with such adverse effects by using a combination of various agents with CsA [34]. The overproduction of superoxide anion and the impairment in the conversion of hydrogen peroxide may contribute to the increased oxidant damages in CsA-treated rats [35].

Melatonin has been reported to have antioxidant properties [6,8,35], but clinical investigation of melatonin agonists has been hampered by side effects such as hypothermia, hypotension and bradycardia [36]. An inappropriate time schedule of melatonin administration could induce supraphysiological concentrations of the neurohormone and a desensitization of melatonin receptors. A long duration of exposure to melatonin also could mimic an “artificial darkness” condition when a circadian rhythm with a basal zero level during the day needs to be conserved for a physiological function. Furthermore, administration of large doses of melatonin could induce high concentrations of melatonin and of different metabolites that could have deleterious effects *per se*. Very little attention has been paid to the possible side effects of melatonin—nightmares, hypotension, sleep disorders and abdominal pain have been reported [37]. In the light of its physiological role in animals, the potential deleterious effects include inhibition of reproductive function, delayed timing of puberty, and influence (when taken during pregnancy and lactation) on the circadian status of the fetus and neonate and on future development. Of its significant short-term side effects in human, sleepiness following oral ingestion of synthetic melatonin has been also reported [38,39]. According to the adverse effects of exogenous melatonin usage, we decided to utilize a combination to stimulate endogenous melatonin together with CsA, to take advantage of anti-oxidative property of melatonin avoiding its exogenous adverse effects. Isoproterenol is reported as one of compounds able to produce endogenous melatonin [40,41]. Isoproterenol is able to stimulate both young and old rat pineal glands no matter what the circadian stage they are in [40]. 

Administration of isoproterenol solely (in group B rats) did not make significant changes in total antioxidant capacity and LDL susceptibility to oxidation. So, isoproterenol administration, with this dosage, neither has a protective effect nor a toxic effect on the measured indices. In our previous study, we showed that administration of isoproterenol (20 mg/kg/d for 21 days) did not cause a significant deterioration in renal function, as well [42]. 

Administration of only CsA decreased the total antioxidant capacity and increased LDL susceptibility to oxidation in group C rats, but administration of CsA plus isoproterenol simultaneously (in group D rats) did not make significant changes in total antioxidant capacity and LDL susceptibility to oxidation, but caused a significant increase in the mean serum melatonin level, which demonstrated that isoproterenol had a protective effect on the deterioration of total antioxidant capacity and LDL susceptibility to oxidation induced by CsA, probably because of its ability to increase production of endogenous melatonin with antioxidant properties.

One week after the treatment in group C rats (which had received only CsA), LDL susceptibility to oxidation was still higher, and the total antioxidant capacity was still lower than their initial levels before CsA administration, which demonstrated that deterioration in mentioned indices was partially irreversible—at least until one week after discontinuation of the drug. Nonetheless, the effect of isoproterenol on the serum melatonin level is reversible at this period after discontinuation of the drug. 

We conclude that isoproterenol stimulates endogenous melatonin production, and prevents the increase in LDL susceptibility to oxidation and decrease in serum total antioxidant capacity in CsA-treated rats. So, isoproterenol may be capable of delaying CsA-induced adverse effects by preventing the deterioration of the measured indices by means of endogenous melatonin (a potential antioxidant agent) production.
